# PP37 Guidance On Using Hospital-Based Real-World Evidence In Health Technology Assessments For Oncology

**DOI:** 10.1017/S0266462324002095

**Published:** 2025-01-07

**Authors:** Zainab Al-khayat, Nora Franzen, Valesca P Retèl, Wim H van Harten

## Abstract

**Introduction:**

Real-world evidence (RWE) is increasingly used in healthcare research to address evidence gaps, reduce uncertainty about medical technology benefits, and provide real-world insights. Efforts to integrate RWE in regulatory and health technology assessment (HTA) processes are growing. However, variations among countries pose challenges. The objective is to analyze and compare various (inter)national RWE guidelines, focusing on real-world hospital data utilization.

**Methods:**

We conducted a review to identify RWE guidance published from 2016 to 2023, with a focus on the EU5 nations (UK, France, Germany, Italy, and Spain) and the ONCOVALUE consortium affiliates (Finland, the Netherlands, Denmark, Italy, and Portugal). To ensure a comprehensive overview, we also investigated Canada, the European Medicines Agency (EMA), ISPOR, and the European Society for Medical Oncology (ESMO). We conducted in-depth interviews with HTA experts of all included countries, focusing on real-world hospital data within the European HTA context. The interviews underwent thematic analysis related to the utilization of RWE in HTA.

**Results:**

We identified nine guidance reports: six focused on HTA-RWE (Medicinrådet/Denmark, NICE/UK, AQuAS/Spain, HAS/France, IQWiG/Germany, CADTH/Canada), one from EMA, and two international (ISPOR, ESMO). Only NICE, IQWiG, and CADTH offered recommendations covering hospital data, emphasizing the data curation process. HAS addressed considerations in choosing secondary data sources, while IQWiG established robust criteria for registries to ensure data quality. Regarding patient-reported outcomes data, only HAS and NICE provided recommendations in their guidance. The HTA experts acknowledged the value of hospital data but expressed caution due to its unstructured nature, noting that the use of hospital-based RWE is more accepted in descriptive studies.

**Conclusions:**

Guidances prioritize the clinical domain, emphasizing transparency, fitness for purpose, reproducibility, robustness, bias minimization, and generalizability. Notably, there’s a lack of comprehensive source-specific guidance for real-world data sources, including registries, hospitals, claims, and wearables. Enhanced guidance on the total data generation process, (data mapping, data federation), cost data, quality of life, and cross-border data usage would strengthen hospital-based RWE assessments.

